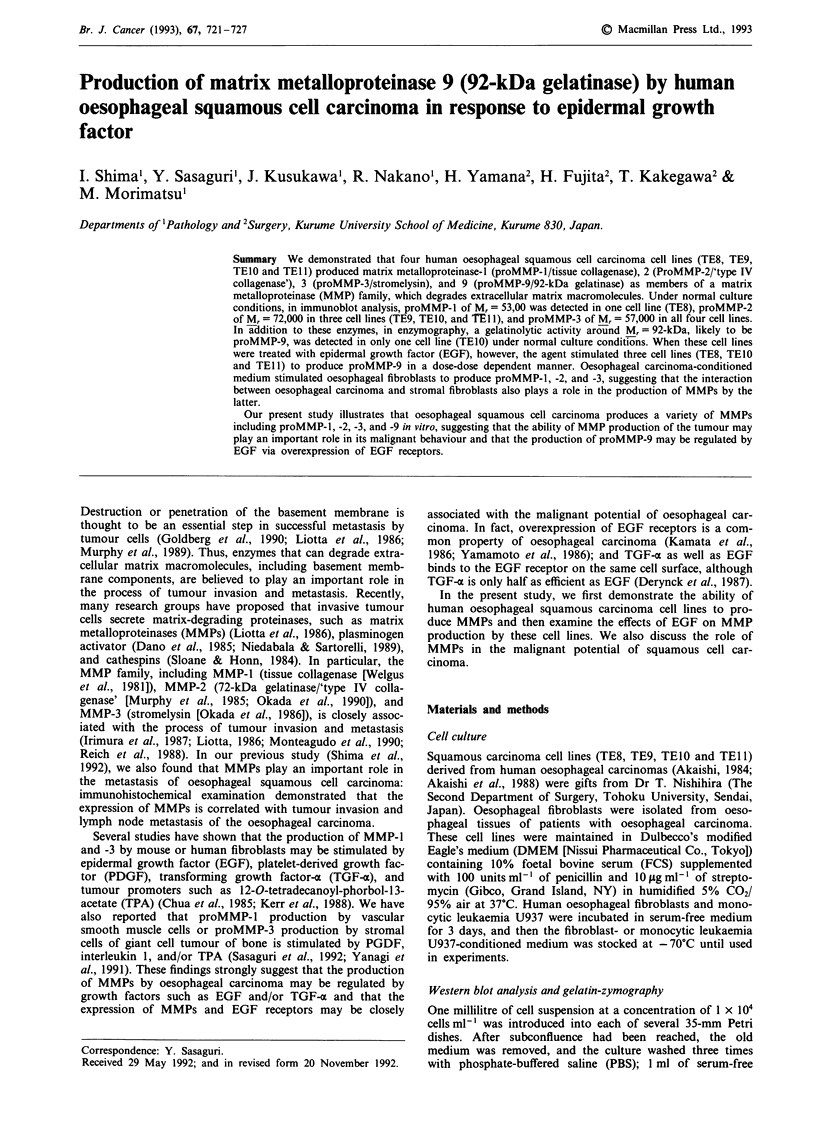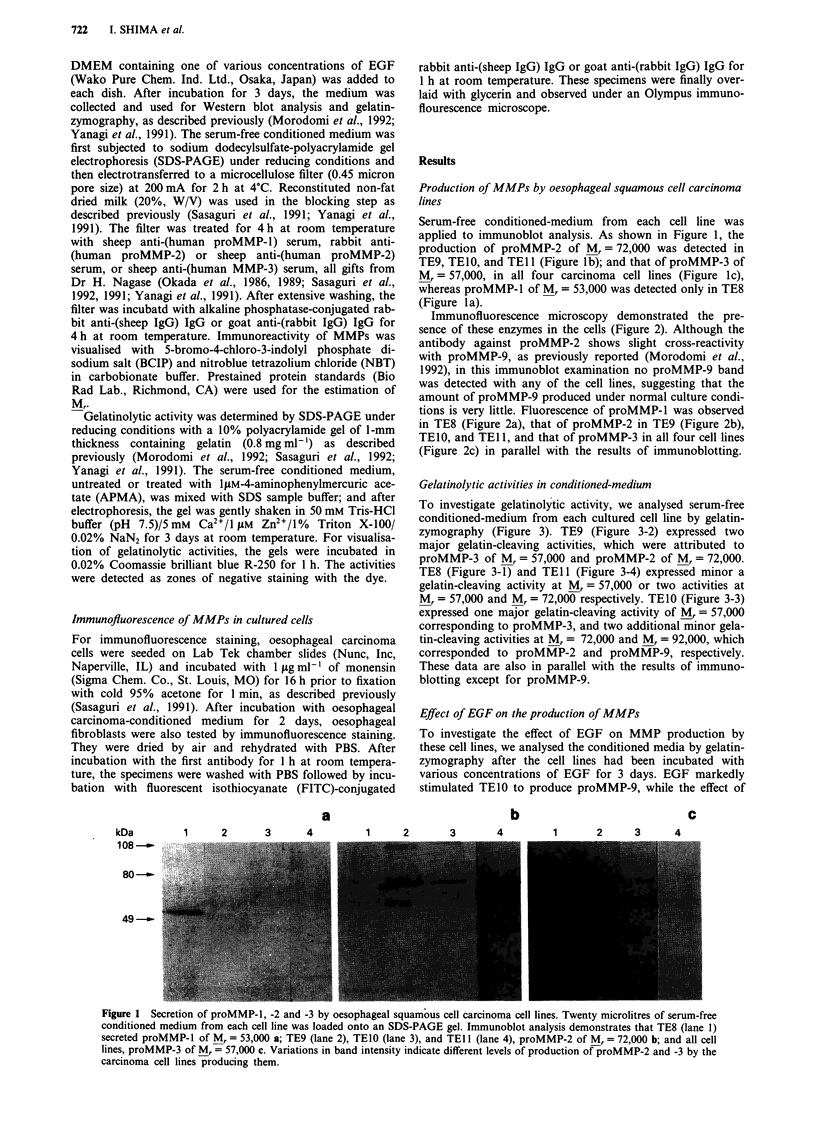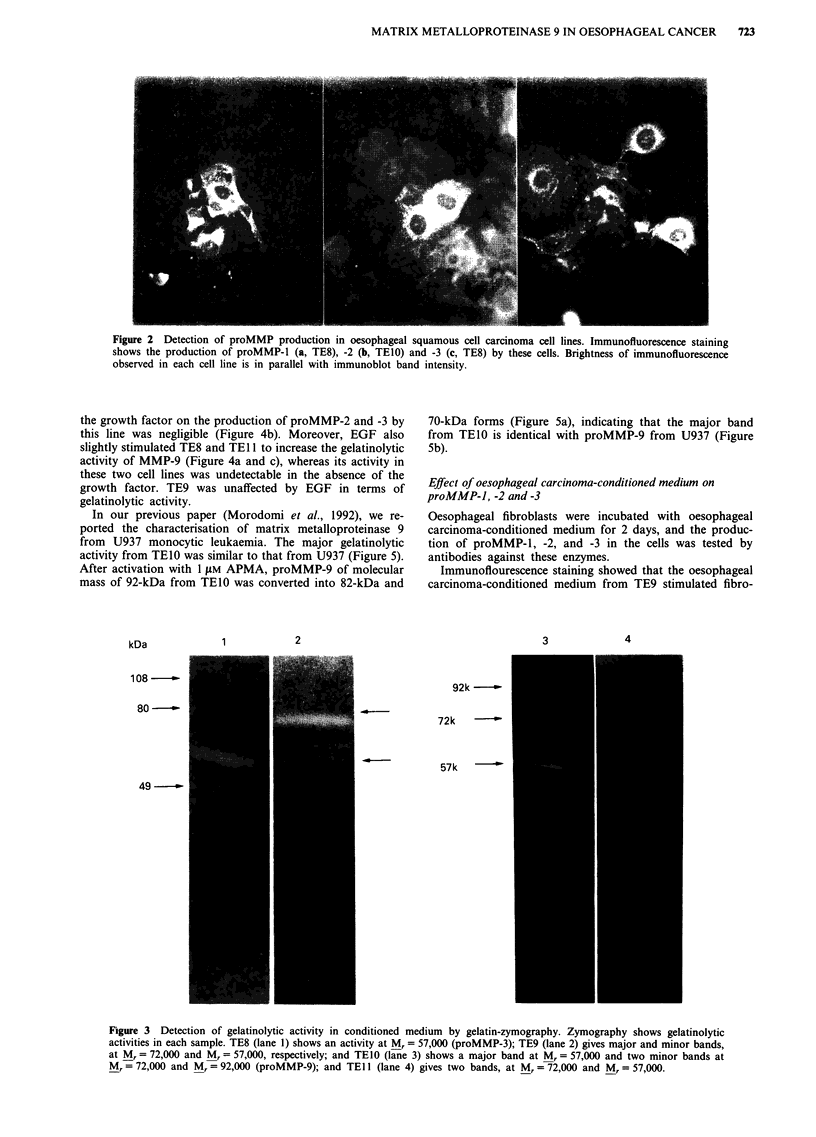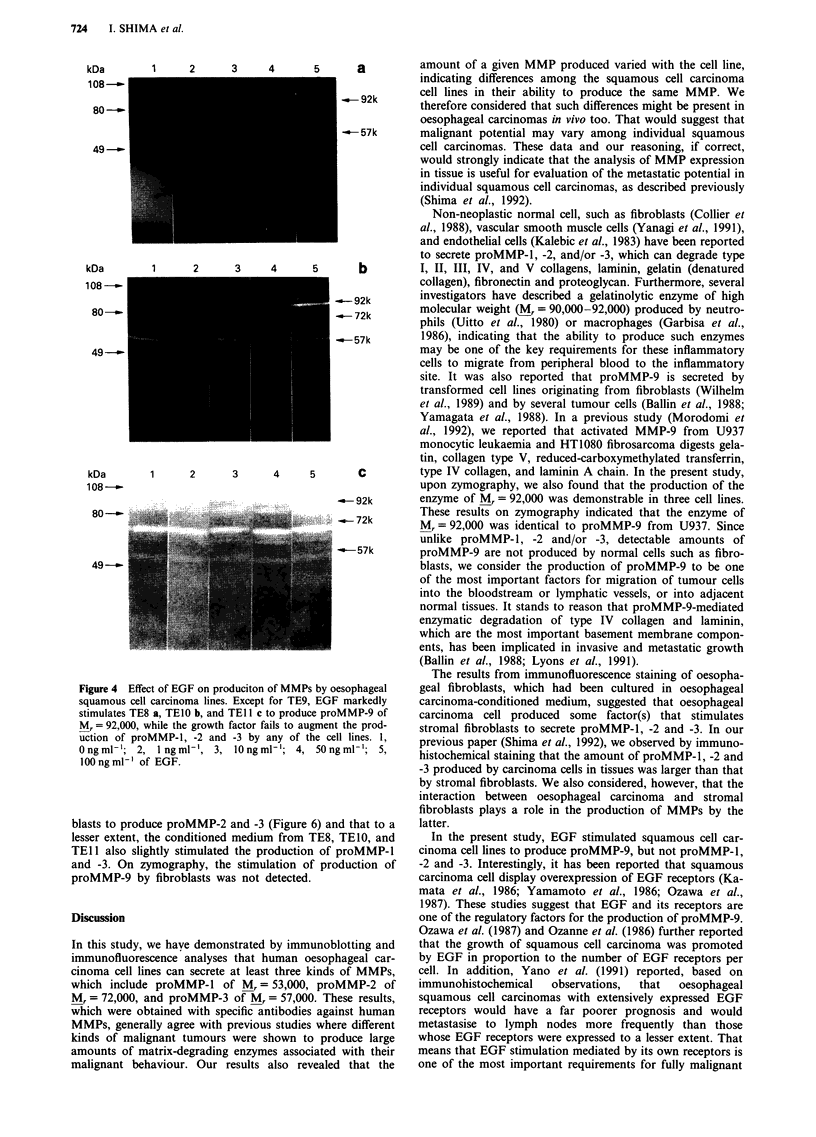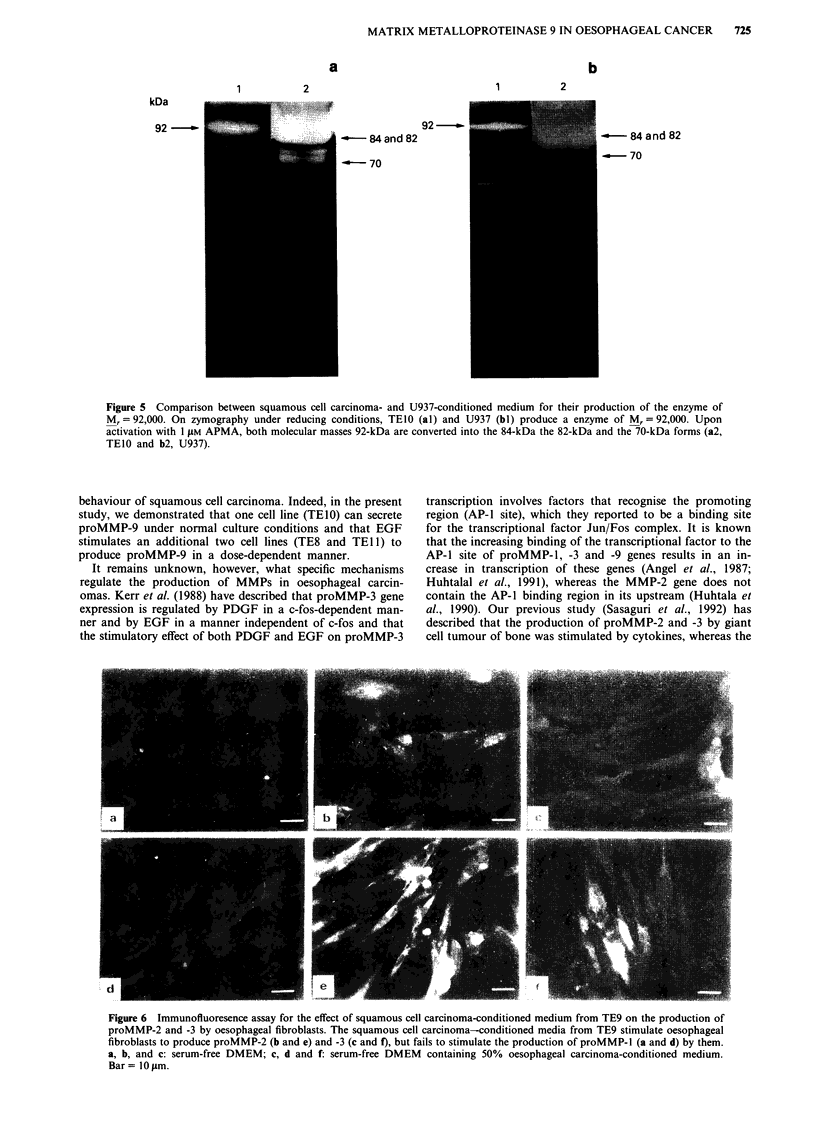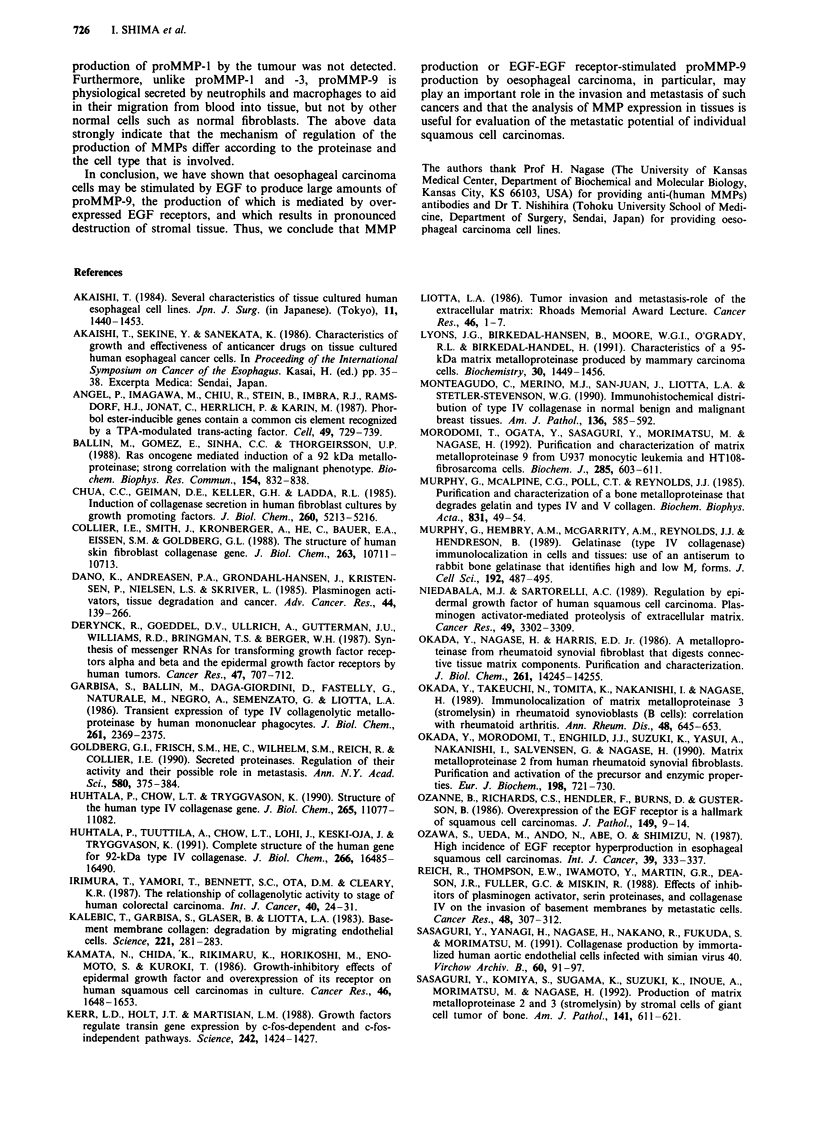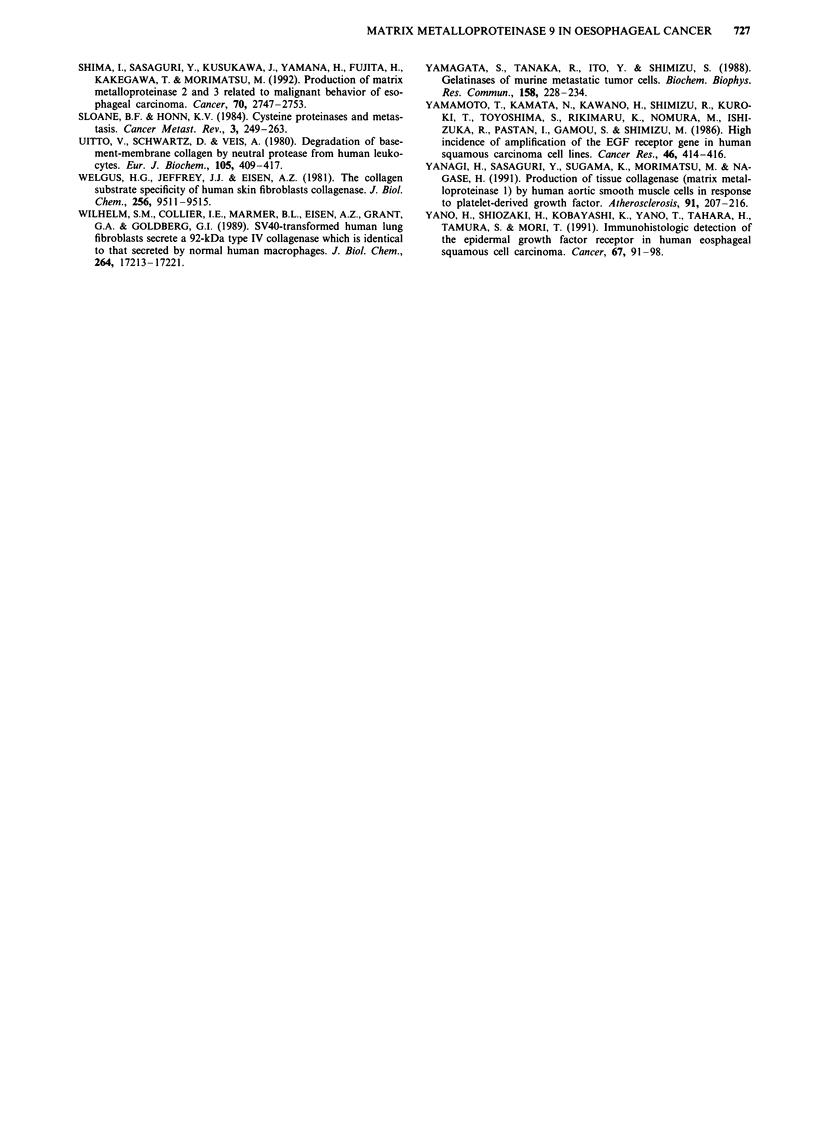# Production of matrix metalloproteinase 9 (92-kDa gelatinase) by human oesophageal squamous cell carcinoma in response to epidermal growth factor.

**DOI:** 10.1038/bjc.1993.132

**Published:** 1993-04

**Authors:** I. Shima, Y. Sasaguri, J. Kusukawa, R. Nakano, H. Yamana, H. Fujita, T. Kakegawa, M. Morimatsu

**Affiliations:** Department of Pathology, Kurume University School of Medicine, Japan.

## Abstract

**Images:**


Br. J. Cancer (1993), 67, 721–727 © Macmillan Press Ltd., 1993

### Production of matrix metalloproteinase 9 (92-kDa gelatinase) by human oesophageal squamous cell carcinoma in response to epidermal growth factor

I. Shima[1], Y. Sasaguri[1], J. Kusukawa[1], R. Nakano[1], H. Yamana[2], H. Fujita[2], T. Kakegawa[2] & M. Morimatsu[1]

*Departments of [1]Pathology and [2]Surgery, Kurume University School of Medicine, Kurume 830, Japan.*

**Summary** We demonstrated that four human oesophageal squamous cell carcinoma cell lines (TE8, TE9, TE10 and TE11) produced matrix metalloproteinase-1 (proMMP-1/tissue collagenase), 2 (ProMMP-2/'type IV collagenase'), 3 (proMMP-3/stromelysin), and 9 (proMMP-9/92-kDa gelatinase) as members of a matrix metalloproteinase (MMP) family, which degrades extracellular matrix macromolecules. Under normal culture conditions, in immunoblot analysis, proMMP-1 of $M_r = 53{,}00$ was detected in one cell line (TE8), proMMP-2 of $M_r = 72{,}000$ in three cell lines (TE9, TE10, and TE11), and proMMP-3 of $M_r = 57{,}000$ in all four cell lines. In addition to these enzymes, in enzymography, a gelatinolytic activity around $M_r = 92$-kDa, likely to be proMMP-9, was detected in only one cell line (TE10) under normal culture conditions. When these cell lines were treated with epidermal growth factor (EGF), however, the agent stimulated three cell lines (TE8, TE10 and TE11) to produce proMMP-9 in a dose-dose dependent manner. Oesophageal carcinoma-conditioned medium stimulated oesophageal fibroblasts to produce proMMP-1, -2, and -3, suggesting that the interaction between oesophageal carcinoma and stromal fibroblasts also plays a role in the production of MMPs by the latter.

Our present study illustrates that oesophageal squamous cell carcinoma produces a variety of MMPs including proMMP-1, -2, -3, and -9 *in vitro*, suggesting that the ability of MMP production of the tumour may play an important role in its malignant behaviour and that the production of proMMP-9 may be regulated by EGF via overexpression of EGF receptors.

Destruction or penetration of the basement membrane is thought to be an essential step in successful metastasis by tumour cells (Goldberg *et al.*, 1990; Liotta *et al.*, 1986; Murphy *et al.*, 1989). Thus, enzymes that can degrade extracellular matrix macromolecules, including basement membrane components, are believed to play an important role in the process of tumour invasion and metastasis. Recently, many research groups have proposed that invasive tumour cells secrete matrix-degrading proteinases, such as matrix metalloproteinases (MMPs) (Liotta *et al.*, 1986), plasminogen activator (Dano *et al.*, 1985; Niedabala & Sartorelli, 1989), and cathespins (Sloane & Honn, 1984). In particular, the MMP family, including MMP-1 (tissue collagenase [Welgus *et al.*, 1981]), MMP-2 (72-kDa gelatinase/'type IV collagenase' [Murphy *et al.*, 1985; Okada *et al.*, 1990]), and MMP-3 (stromelysin [Okada *et al.*, 1986]), is closely associated with the process of tumour invasion and metastasis (Irimura *et al.*, 1987; Liotta, 1986; Monteagudo *et al.*, 1990; Reich *et al.*, 1988). In our previous study (Shima *et al.*, 1992), we also found that MMPs play an important role in the metastasis of oesophageal squamous cell carcinoma: immunohistochemical examination demonstrated that the expression of MMPs is correlated with tumour invasion and lymph node metastasis of the oesophageal carcinoma.

Several studies have shown that the production of MMP-1 and -3 by mouse or human fibroblasts may be stimulated by epidermal growth factor (EGF), platelet-derived growth factor (PDGF), transforming growth factor-α (TGF-α), and tumour promoters such as 12-*O*-tetradecanoyl-phorbol-13-acetate (TPA) (Chua *et al.*, 1985; Kerr *et al.*, 1988). We have also reported that proMMP-1 production by vascular smooth muscle cells or proMMP-3 production by stromal cells of giant cell tumour of bone is stimulated by PGDF, interleukin 1, and/or TPA (Sasaguri *et al.*, 1992; Yanagi *et al.*, 1991). These findings strongly suggest that the production of MMPs by oesophageal carcinoma may be regulated by growth factors such as EGF and/or TGF-α and that the expression of MMPs and EGF receptors may be closely associated with the malignant potential of oesophageal carcinoma. In fact, overexpression of EGF receptors is a common property of oesophageal carcinoma (Kamata *et al.*, 1986; Yamamoto *et al.*, 1986); and TGF-α as well as EGF binds to the EGF receptor on the same cell surface, although TGF-α is only half as efficient as EGF (Derynck *et al.*, 1987).

In the present study, we first demonstrate the ability of human oesophageal squamous carcinoma cell lines to produce MMPs and then examine the effects of EGF on MMP production by these cell lines. We also discuss the role of MMPs in the malignant potential of squamous cell carcinoma.

Correspondence: Y. Sasaguri.
Received 29 May 1992; and in revised form 20 November 1992.

### Materials and methods

### *Cell culture*

Squamous carcinoma cell lines (TE8, TE9, TE10 and TE11) derived from human oesophageal carcinomas (Akaishi, 1984; Akaishi *et al.*, 1988) were gifts from Dr T. Nishihira (The Second Department of Surgery, Tohoku University, Sendai, Japan). Oesophageal fibroblasts were isolated from oesophageal tissues of patients with oesophageal carcinoma. These cell lines were maintained in Dulbecco's modified Eagle's medium (DMEM [Nissui Pharmaceutical Co., Tokyo]) containing 10% foetal bovine serum (FCS) supplemented with 100 units $ml^{-1}$ of penicillin and 10 $\mu g\ ml^{-1}$ of streptomycin (Gibco, Grand Island, NY) in humidified 5% $CO_2$/95% air at 37°C. Human oesophageal fibroblasts and monocytic leukaemia U937 were incubated in serum-free medium for 3 days, and then the fibroblast- or monocytic leukaemia U937-conditioned medium was stocked at −70°C until used in experiments.

### *Western blot analysis and gelatin-zymography*

One millilitre of cell suspension at a concentration of $1 \times 10^4$ cells $ml^{-1}$ was introduced into each of several 35-mm Petri dishes. After subconfluence had been reached, the old medium was removed, and the culture washed three times with phosphate-buffered saline (PBS); 1 ml of serum-free

DMEM containing one of various concentrations of EGF (Wako Pure Chem. Ind. Ltd., Osaka, Japan) was added to each dish. After incubation for 3 days, the medium was collected and used for Western blot analysis and gelatin-zymography, as described previously (Morodomi *et al.*, 1992; Yanagi *et al.*, 1991). The serum-free conditioned medium was first subjected to sodium dodecylsulfate-polyacrylamide gel electrophoresis (SDS-PAGE) under reducing conditions and then electrotransferred to a microcellulose filter (0.45 micron pore size) at 200 mA for 2 h at 4°C. Reconstituted non-fat dried milk (20%, W/V) was used in the blocking step as described previously (Sasaguri *et al.*, 1991; Yanagi *et al.*, 1991). The filter was treated for 4 h at room temperature with sheep anti-(human proMMP-1) serum, rabbit anti-(human proMMP-2) or sheep anti-(human proMMP-2) serum, or sheep anti-(human MMP-3) serum, all gifts from Dr H. Nagase (Okada *et al.*, 1986, 1989; Sasaguri *et al.*, 1992, 1991; Yanagi *et al.*, 1991). After extensive washing, the filter was incubatd with alkaline phosphatase-conjugated rabbit anti-(sheep IgG) IgG or goat anti-(rabbit IgG) IgG for 4 h at room temperature. Immunoreactivity of MMPs was visualised with 5-bromo-4-chloro-3-indolyl phosphate disodium salt (BCIP) and nitroblue tetrazolium chloride (NBT) in carbobionate buffer. Prestained protein standards (Bio Rad Lab., Richmond, CA) were used for the estimation of $\underline{M}_r$.

Gelatinolytic activity was determined by SDS-PAGE under reducing conditions with a 10% polyacrylamide gel of 1-mm thickness containing gelatin ($0.8\ mg\ ml^{-1}$) as described previously (Morodomi *et al.*, 1992; Sasaguri *et al.*, 1992; Yanagi *et al.*, 1991). The serum-free conditioned medium, untreated or treated with 1μM-4-aminophenylmercuric acetate (APMA), was mixed with SDS sample buffer; and after electrophoresis, the gel was gently shaken in 50 mM Tris-HCl buffer (pH 7.5)/5 mM $Ca^{2+}$/1 μM $Zn^{2+}$/1% Triton X-100/0.02% $NaN_2$ for 3 days at room temperature. For visualisation of gelatinolytic activities, the gels were incubated in 0.02% Coomassie brilliant blue R-250 for 1 h. The activities were detected as zones of negative staining with the dye.

### *Immunofluorescence of MMPs in cultured cells*

For immunofluorescence staining, oesophageal carcinoma cells were seeded on Lab Tek chamber slides (Nunc, Inc, Naperville, IL) and incubated with $1\ \mu g\ ml^{-1}$ of monensin (Sigma Chem. Co., St. Louis, MO) for 16 h prior to fixation with cold 95% acetone for 1 min, as described previously (Sasaguri *et al.*, 1991). After incubation with oesophageal carcinoma-conditioned medium for 2 days, oesophageal fibroblasts were also tested by immunofluorescence staining. They were dried by air and rehydrated with PBS. After incubation with the first antibody for 1 h at room temperature, the specimens were washed with PBS followed by incubation with fluorescent isothiocyanate (FITC)-conjugated rabbit anti-(sheep IgG) IgG or goat anti-(rabbit IgG) IgG for 1 h at room temperature. These specimens were finally overlaid with glycerin and observed under an Olympus immunoflourescence microscope.

### Results

### *Production of MMPs by oesophageal squamous cell carcinoma lines*

Serum-free conditioned-medium from each cell line was applied to immunoblot analysis. As shown in Figure 1, the production of proMMP-2 of $\underline{M}_r = 72,000$ was detected in TE9, TE10, and TE11 (Figure 1b); and that of proMMP-3 of $\underline{M}_r = 57,000$, in all four carcinoma cell lines (Figure 1c), whereas proMMP-1 of $\underline{M}_r = 53,000$ was detected only in TE8 (Figure 1a).

Immunofluorescence microscopy demonstrated the presence of these enzymes in the cells (Figure 2). Although the antibody against proMMP-2 shows slight cross-reactivity with proMMP-9, as previously reported (Morodomi *et al.*, 1992), in this immunoblot examination no proMMP-9 band was detected with any of the cell lines, suggesting that the amount of proMMP-9 produced under normal culture conditions is very little. Fluorescence of proMMP-1 was observed in TE8 (Figure 2a), that of proMMP-2 in TE9 (Figure 2b), TE10, and TE11, and that of proMMP-3 in all four cell lines (Figure 2c) in parallel with the results of immunoblotting.

### *Gelatinolytic activities in conditioned-medium*

To investigate gelatinolytic activity, we analysed serum-free conditioned-medium from each cultured cell line by gelatin-zymography (Figure 3). TE9 (Figure 3-2) expressed two major gelatin-cleaving activities, which were attributed to proMMP-3 of $\underline{M}_r = 57,000$ and proMMP-2 of $\underline{M}_r = 72,000$. TE8 (Figure 3-1) and TE11 (Figure 3-4) expressed minor a gelatin-cleaving activity at $\underline{M}_r = 57,000$ or two activities at $\underline{M}_r = 57,000$ and $\underline{M}_r = 72,000$ respectively. TE10 (Figure 3-3) expressed one major gelatin-cleaving activity of $\underline{M}_r = 57,000$ corresponding to proMMP-3, and two additional minor gelatin-cleaving activities at $\underline{M}_r = 72,000$ and $\underline{M}_r = 92,000$, which corresponded to proMMP-2 and proMMP-9, respectively. These data are also in parallel with the results of immunoblotting except for proMMP-9.

### *Effect of EGF on the production of MMPs*

To investigate the effect of EGF on MMP production by these cell lines, we analysed the conditioned media by gelatin-zymography after the cell lines had been incubated with various concentrations of EGF for 3 days. EGF markedly stimulated TE10 to produce proMMP-9, while the effect of

**Figure 1** Secretion of proMMP-1, -2 and -3 by oesophageal squamous cell carcinoma cell lines. Twenty microlitres of serum-free conditioned medium from each cell line was loaded onto an SDS-PAGE gel. Immunoblot analysis demonstrates that TE8 (lane 1) secreted proMMP-1 of $\underline{M}_r = 53,000$ **a**; TE9 (lane 2), TE10 (lane 3), and TE11 (lane 4), proMMP-2 of $\underline{M}_r = 72,000$ **b**; and all cell lines, proMMP-3 of $\underline{M}_r = 57,000$ **c**. Variations in band intensity indicate different levels of production of proMMP-2 and -3 by the carcinoma cell lines producing them.

**Figure 2** Detection of proMMP production in oesophageal squamous cell carcinoma cell lines. Immunofluorescence staining shows the production of proMMP-1 (**a**, TE8), -2 (**b**, TE10) and -3 (**c**, TE8) by these cells. Brightness of immunofluorescence observed in each cell line is in parallel with immunoblot band intensity.

the growth factor on the production of proMMP-2 and -3 by this line was negligible (Figure 4b). Moreover, EGF also slightly stimulated TE8 and TE11 to increase the gelatinolytic activity of MMP-9 (Figure 4a and c), whereas its activity in these two cell lines was undetectable in the absence of the growth factor. TE9 was unaffected by EGF in terms of gelatinolytic activity.

In our previous paper (Morodomi *et al.*, 1992), we reported the characterisation of matrix metalloproteinase 9 from U937 monocytic leukaemia. The major gelatinolytic activity from TE10 was similar to that from U937 (Figure 5). After activation with 1 μM APMA, proMMP-9 of molecular mass of 92-kDa from TE10 was converted into 82-kDa and 70-kDa forms (Figure 5a), indicating that the major band from TE10 is identical with proMMP-9 from U937 (Figure 5b).

*Effect of oesophageal carcinoma-conditioned medium on proMMP-1, -2 and -3*

Oesophageal fibroblasts were incubated with oesophageal carcinoma-conditioned medium for 2 days, and the production of proMMP-1, -2, and -3 in the cells was tested by antibodies against these enzymes.

Immunoflourescence staining showed that the oesophageal carcinoma-conditioned medium from TE9 stimulated fibro-

**Figure 3** Detection of gelatinolytic activity in conditioned medium by gelatin-zymography. Zymography shows gelatinolytic activities in each sample. TE8 (lane 1) shows an activity at $M_r$ = 57,000 (proMMP-3); TE9 (lane 2) gives major and minor bands, at $M_r$ = 72,000 and $M_r$ = 57,000, respectively; and TE10 (lane 3) shows a major band at $M_r$ = 57,000 and two minor bands at $M_r$ = 72,000 and $M_r$ = 92,000 (proMMP-9); and TE11 (lane 4) gives two bands, at $M_r$ = 72,000 and $M_r$ = 57,000.

**Figure 4** Effect of EGF on produciton of MMPs by oesophageal squamous cell carcinoma lines. Except for TE9, EGF markedly stimulates TE8 **a**, TE10 **b**, and TE11 **c** to produce proMMP-9 of $M_r$ = 92,000, while the growth factor fails to augment the production of proMMP-1, -2 and -3 by any of the cell lines. 1, 0 ng ml$^{-1}$; 2, 1 ng ml$^{-1}$, 3, 10 ng ml$^{-1}$; 4, 50 ng ml$^{-1}$; 5, 100 ng ml$^{-1}$ of EGF.

blasts to produce proMMP-2 and -3 (Figure 6) and that to a lesser extent, the conditioned medium from TE8, TE10, and TE11 also slightly stimulated the production of proMMP-1 and -3. On zymography, the stimulation of production of proMMP-9 by fibroblasts was not detected.

### Discussion

In this study, we have demonstrated by immunoblotting and immunofluorescence analyses that human oesophageal carcinoma cell lines can secrete at least three kinds of MMPs, which include proMMP-1 of $M_r$ = 53,000, proMMP-2 of $M_r$ = 72,000, and proMMP-3 of $M_r$ = 57,000. These results, which were obtained with specific antibodies against human MMPs, generally agree with previous studies where different kinds of malignant tumours were shown to produce large amounts of matrix-degrading enzymes associated with their malignant behaviour. Our results also revealed that the amount of a given MMP produced varied with the cell line, indicating differences among the squamous cell carcinoma cell lines in their ability to produce the same MMP. We therefore considered that such differences might be present in oesophageal carcinomas *in vivo* too. That would suggest that malignant potential may vary among individual squamous cell carcinomas. These data and our reasoning, if correct, would strongly indicate that the analysis of MMP expression in tissue is useful for evaluation of the metastatic potential in individual squamous cell carcinomas, as described previously (Shima *et al.*, 1992).

Non-neoplastic normal cell, such as fibroblasts (Collier *et al.*, 1988), vascular smooth muscle cells (Yanagi *et al.*, 1991), and endothelial cells (Kalebic *et al.*, 1983) have been reported to secrete proMMP-1, -2, and/or -3, which can degrade type I, II, III, IV, and V collagens, laminin, gelatin (denatured collagen), fibronectin and proteoglycan. Furthermore, several investigators have described a gelatinolytic enzyme of high molecular weight ($M_r$ = 90,000–92,000) produced by neutrophils (Uitto *et al.*, 1980) or macrophages (Garbisa *et al.*, 1986), indicating that the ability to produce such enzymes may be one of the key requirements for these inflammatory cells to migrate from peripheral blood to the inflammatory site. It was also reported that proMMP-9 is secreted by transformed cell lines originating from fibroblasts (Wilhelm *et al.*, 1989) and by several tumour cells (Ballin *et al.*, 1988; Yamagata *et al.*, 1988). In a previous study (Morodomi *et al.*, 1992), we reported that activated MMP-9 from U937 monocytic leukaemia and HT1080 fibrosarcoma digests gelatin, collagen type V, reduced-carboxymethylated transferrin, type IV collagen, and laminin A chain. In the present study, upon zymography, we also found that the production of the enzyme of $M_r$ = 92,000 was demonstrable in three cell lines. These results on zymography indicated that the enzyme of $M_r$ = 92,000 was identical to proMMP-9 from U937. Since unlike proMMP-1, -2 and/or -3, detectable amounts of proMMP-9 are not produced by normal cells such as fibroblasts, we consider the production of proMMP-9 to be one of the most important factors for migration of tumour cells into the bloodstream or lymphatic vessels, or into adjacent normal tissues. It stands to reason that proMMP-9-mediated enzymatic degradation of type IV collagen and laminin, which are the most important basement membrane components, has been implicated in invasive and metastatic growth (Ballin *et al.*, 1988; Lyons *et al.*, 1991).

The results from immunofluorescence staining of oesophageal fibroblasts, which had been cultured in oesophageal carcinoma-conditioned medium, suggested that oesophageal carcinoma cell produced some factor(s) that stimulates stromal fibroblasts to secrete proMMP-1, -2 and -3. In our previous paper (Shima *et al.*, 1992), we observed by immunohistochemical staining that the amount of proMMP-1, -2 and -3 produced by carcinoma cells in tissues was larger than that by stromal fibroblasts. We also considered, however, that the interaction between oesophageal carcinoma and stromal fibroblasts plays a role in the production of MMPs by the latter.

In the present study, EGF stimulated squamous cell carcinoma cell lines to produce proMMP-9, but not proMMP-1, -2 and -3. Interestingly, it has been reported that squamous carcinoma cell display overexpression of EGF receptors (Kamata *et al.*, 1986; Yamamoto *et al.*, 1986; Ozawa *et al.*, 1987). These studies suggest that EGF and its receptors are one of the regulatory factors for the production of proMMP-9. Ozawa *et al.* (1987) and Ozanne *et al.* (1986) further reported that the growth of squamous cell carcinoma was promoted by EGF in proportion to the number of EGF receptors per cell. In addition, Yano *et al.* (1991) reported, based on immunohistochemical observations, that oesophageal squamous cell carcinomas with extensively expressed EGF receptors would have a far poorer prognosis and would metastasise to lymph nodes more frequently than those whose EGF receptors were expressed to a lesser extent. That means that EGF stimulation mediated by its own receptors is one of the most important requirements for fully malignant

**Figure 5** Comparison between squamous cell carcinoma- and U937-conditioned medium for their production of the enzyme of $M_r = 92,000$. On zymography under reducing conditions, TE10 (**a**1) and U937 (**b**1) produce a enzyme of $M_r = 92,000$. Upon activation with 1 μM APMA, both molecular masses 92-kDa are converted into the 84-kDa the 82-kDa and the 70-kDa forms (**a**2, TE10 and **b**2, U937).

behaviour of squamous cell carcinoma. Indeed, in the present study, we demonstrated that one cell line (TE10) can secrete proMMP-9 under normal culture conditions and that EGF stimulates an additional two cell lines (TE8 and TE11) to produce proMMP-9 in a dose-dependent manner.

It remains unknown, however, what specific mechanisms regulate the production of MMPs in oesophageal carcinomas. Kerr *et al.* (1988) have described that proMMP-3 gene expression is regulated by PDGF in a c-fos-dependent manner and by EGF in a manner independent of c-fos and that the stimulatory effect of both PDGF and EGF on proMMP-3 transcription involves factors that recognise the promoting region (AP-1 site), which they reported to be a binding site for the transcriptional factor Jun/Fos complex. It is known that the increasing binding of the transcriptional factor to the AP-1 site of proMMP-1, -3 and -9 genes results in an increase in transcription of these genes (Angel *et al.*, 1987; Huhtalal *et al.*, 1991), whereas the MMP-2 gene does not contain the AP-1 binding region in its upstream (Huhtala *et al.*, 1990). Our previous study (Sasaguri *et al.*, 1992) has described that the production of proMMP-2 and -3 by giant cell tumour of bone was stimulated by cytokines, whereas the

**Figure 6** Immunofluoresence assay for the effect of squamous cell carcinoma-conditioned medium from TE9 on the production of proMMP-2 and -3 by oesophageal fibroblasts. The squamous cell carcinoma–conditioned media from TE9 stimulate oesophageal fibroblasts to produce proMMP-2 (**b** and **e**) and -3 (**c** and **f**), but fails to stimulate the production of proMMP-1 (**a** and **d**) by them. **a**, **b**, and **c**: serum-free DMEM; **c**, **d** and **f**: serum-free DMEM containing 50% oesophageal carcinoma-conditioned medium. Bar = 10 μm.

production of proMMP-1 by the tumour was not detected. Furthermore, unlike proMMP-1 and -3, proMMP-9 is physiological secreted by neutrophils and macrophages to aid in their migration from blood into tissue, but not by other normal cells such as normal fibroblasts. The above data strongly indicate that the mechanism of regulation of the production of MMPs differ according to the proteinase and the cell type that is involved.

In conclusion, we have shown that oesophageal carcinoma cells may be stimulated by EGF to produce large amounts of proMMP-9, the production of which is mediated by over-expressed EGF receptors, and which results in pronounced destruction of stromal tissue. Thus, we conclude that MMP production or EGF-EGF receptor-stimulated proMMP-9 production by oesophageal carcinoma, in particular, may play an important role in the invasion and metastasis of such cancers and that the analysis of MMP expression in tissues is useful for evaluation of the metastatic potential of individual squamous cell carcinomas.

The authors thank Prof H. Nagase (The University of Kansas Medical Center, Department of Biochemical and Molecular Biology, Kansas City, KS 66103, USA) for providing anti-(human MMPs) antibodies and Dr T. Nishihira (Tohoku University School of Medicine, Department of Surgery, Sendai, Japan) for providing oesophageal carcinoma cell lines.

### References

AKAISHI, T. (1984). Several characteristics of tissue cultured human esophageal cell lines. *Jpn. J. Surg.* (in Japanese). (Tokyo), **11**, 1440–1453.

AKAISHI, T., SEKINE, Y. & SANEKATA, K. (1986). Characteristics of growth and effectiveness of anticancer drugs on tissue cultured human esophageal cancer cells. In *Proceeding of the International Symposium on Cancer of the Esophagus*. Kasai, H. (ed.) pp. 35–38. Excerpta Medica: Sendai, Japan.

ANGEL, P., IMAGAWA, M., CHIU, R., STEIN, B., IMBRA, R.J., RAMSDORF, H.J., JONAT, C., HERRLICH, P. & KARIN, M. (1987). Phorbol ester-inducible genes contain a common cis element recognized by a TPA-modulated trans-acting factor. *Cell*, **49**, 729–739.

BALLIN, M., GOMEZ, E., SINHA, C.C. & THORGEIRSSON, U.P. (1988). Ras oncogene mediated induction of a 92 kDa metalloproteinase; strong correlation with the malignant phenotype. *Biochem. Biophys. Res. Commun.*, **154**, 832–838.

CHUA, C.C., GEIMAN, D.E., KELLER, G.H. & LADDA, R.L. (1985). Induction of collagenase secretion in human fibroblast cultures by growth promoting factors. *J. Biol. Chem.*, **260**, 5213–5216.

COLLIER, I.E., SMITH, J., KRONBERGER, A., HE, C., BAUER, E.A., EISSEN, S.M. & GOLDBERG, G.L. (1988). The structure of human skin fibroblast collagenase gene. *J. Biol. Chem.*, **263**, 10711–10713.

DANO, K., ANDREASEN, P.A., GRONDAHL-HANSEN, J., KRISTENSEN, P., NIELSEN, L.S. & SKRIVER, L. (1985). Plasminogen activators, tissue degradation and cancer. *Adv. Cancer. Res.*, **44**, 139–266.

DERYNCK, R., GOEDDEL, D.V., ULLRICH, A., GUTTERMAN, J.U., WILLIAMS, R.D., BRINGMAN, T.S. & BERGER, W.H. (1987). Synthesis of messenger RNAs for transforming growth factor receptors alpha and beta and the epidermal growth factor receptors by human tumors. *Cancer Res.*, **47**, 707–712.

GARBISA, S., BALLIN, M., DAGA-GIORDINI, D., FASTELLY, G., NATURALE, M., NEGRO, A., SEMENZATO, G. & LIOTTA, L.A. (1986). Transient expression of type IV collagenolytic metalloproteinase by human mononuclear phagocytes. *J. Biol. Chem.*, **261**, 2369–2375.

GOLDBERG, G.I., FRISCH, S.M., HE, C., WILHELM, S.M., REICH, R. & COLLIER, I.E. (1990). Secreted proteinases. Regulation of their activity and their possible role in metastasis. *Ann. N.Y. Acad. Sci.*, **580**, 375–384.

HUHTALA, P., CHOW, L.T. & TRYGGVASON, K. (1990). Structure of the human type IV collagenase gene. *J. Biol. Chem.*, **265**, 11077–11082.

HUHTALA, P., TUUTTILA, A., CHOW, L.T., LOHI, J., KESKI-OJA, J. & TRYGGVASON, K. (1991). Complete structure of the human gene for 92-kDa type IV collagenase. *J. Biol. Chem.*, **266**, 16485–16490.

IRIMURA, T., YAMORI, T., BENNETT, S.C., OTA, D.M. & CLEARY, K.R. (1987). The relationship of collagenolytic activity to stage of human colorectal carcinoma. *Int. J. Cancer*, **40**, 24–31.

KALEBIC, T., GARBISA, S., GLASER, B. & LIOTTA, L.A. (1983). Basement membrane collagen: degradation by migrating endothelial cells. *Science*, **221**, 281–283.

KAMATA, N., CHIDA, K., RIKIMARU, K., HORIKOSHI, M., ENOMOTO, S. & KUROKI, T. (1986). Growth-inhibitory effects of epidermal growth factor and overexpression of its receptor on human squamous cell carcinomas in culture. *Cancer Res.*, **46**, 1648–1653.

KERR, L.D., HOLT, J.T. & MARTISIAN, L.M. (1988). Growth factors regulate transin gene expression by c-fos-dependent and c-fos-independent pathways. *Science*, **242**, 1424–1427.

LIOTTA, L.A. (1986). Tumor invasion and metastasis-role of the extracellular matrix: Rhoads Memorial Award Lecture. *Cancer Res.*, **46**, 1–7.

LYONS, J.G., BIRKEDAL-HANSEN, B., MOORE, W.G.I., O'GRADY, R.L. & BIRKEDAL-HANDEL, H. (1991). Characteristics of a 95-kDa matrix metalloproteinase produced by mammary carcinoma cells. *Biochemistry*, **30**, 1449–1456.

MONTEAGUDO, C., MERINO, M.J., SAN-JUAN, J., LIOTTA, L.A. & STETLER-STEVENSON, W.G. (1990). Immunohistochemical distribution of type IV collagenase in normal benign and malignant breast tissues. *Am. J. Pathol.*, **136**, 585–592.

MORODOMI, T., OGATA, Y., SASAGURI, Y., MORIMATSU, M. & NAGASE, H. (1992). Purification and characterization of matrix metalloproteinase 9 from U937 monocytic leukemia and HT108-fibrosarcoma cells. *Biochem. J.*, **285**, 603–611.

MURPHY, G., MCALPINE, C.G., POLL, C.T. & REYNOLDS, J.J. (1985). Purification and characterization of a bone metalloproteinase that degrades gelatin and types IV and V collagen. *Biochem. Biophys. Acta.*, **831**, 49–54.

MURPHY, G., HEMBRY, A.M., MCGARRITY, A.M., REYNOLDS, J.J. & HENDRESON, B. (1989). Gelatinase (type IV collagenase) immunolocalization in cells and tissues: use of an antiserum to rabbit bone gelatinase that identifies high and low $M_r$ forms. *J. Cell Sci.*, **192**, 487–495.

NIEDABALA, M.J. & SARTORELLI, A.C. (1989). Regulation by epidermal growth factor of human squamous cell carcinoma. Plasminogen activator-mediated proteolysis of extracellular matrix. *Cancer Res.*, **49**, 3302–3309.

OKADA, Y., NAGASE, H. & HARRIS, E.D. Jr. (1986). A metalloproteinase from rheumatoid synovial fibroblast that digests connective tissue matrix components. Purification and characterization. *J. Biol. Chem.*, **261**, 14245–14255.

OKADA, Y., TAKEUCHI, N., TOMITA, K., NAKANISHI, I. & NAGASE, H. (1989). Immunolocalization of matrix metalloproteinase 3 (stromelysin) in rheumatoid synovioblasts (B cells): correlation with rheumatoid arthritis. *Ann. Rheum. Dis.*, **48**, 645–653.

OKADA, Y., MORODOMI, T., ENGHILD, J.J., SUZUKI, K., YASUI, A., NAKANISHI, I., SALVENSEN, G. & NAGASE, H. (1990). Matrix metalloproteinase 2 from human rheumatoid synovial fibroblasts. Purification and activation of the precursor and enzymic properties. *Eur. J. Biochem.*, **198**, 721–730.

OZANNE, B., RICHARDS, C.S., HENDLER, F., BURNS, D. & GUSTERSON, B. (1986). Overexpression of the EGF receptor is a hallmark of squamous cell carcinomas. *J. Pathol.*, **149**, 9–14.

OZAWA, S., UEDA, M., ANDO, N., ABE, O. & SHIMIZU, N. (1987). High incidence of EGF receptor hyperproduction in esophageal squamous cell carcinomas. *Int. J. Cancer*, **39**, 333–337.

REICH, R., THOMPSON, E.W., IWAMOTO, Y., MARTIN, G.R., DEASON, J.R., FULLER, G.C. & MISKIN, R. (1988). Effects of inhibitors of plasminogen activator, serin proteinases, and collagenase IV on the invasion of basement membranes by metastatic cells. *Cancer Res.*, **48**, 307–312.

SASAGURI, Y., YANAGI, H., NAGASE, H., NAKANO, R., FUKUDA, S. & MORIMATSU, M. (1991). Collagenase production by immortalized human aortic endothelial cells infected with simian virus 40. *Virchow Archiv. B.*, **60**, 91–97.

SASAGURI, Y., KOMIYA, S., SUGAMA, K., SUZUKI, K., INOUE, A., MORIMATSU, M. & NAGASE, H. (1992). Production of matrix metalloproteinase 2 and 3 (stromelysin) by stromal cells of giant cell tumor of bone. *Am. J. Pathol.*, **141**, 611–621.

SHIMA, I., SASAGURI, Y., KUSUKAWA, J., YAMANA, H., FUJITA, H., KAKEGAWA, T. & MORIMATSU, M. (1992). Production of matrix metalloproteinase 2 and 3 related to malignant behavior of esophageal carcinoma. *Cancer*, **70**, 2747–2753.

SLOANE, B.F. & HONN, K.V. (1984). Cysteine proteinases and metastasis. *Cancer Metast. Rev.*, **3**, 249–263.

UITTO, V., SCHWARTZ, D. & VEIS, A. (1980). Degradation of basement-membrane collagen by neutral protease from human leukocytes. *Eur. J. Biochem.*, **105**, 409–417.

WELGUS, H.G., JEFFREY, J.J. & EISEN, A.Z. (1981). The collagen substrate specificity of human skin fibroblasts collagenase. *J. Biol. Chem.*, **256**, 9511–9515.

WILHELM, S.M., COLLIER, I.E., MARMER, B.L., EISEN, A.Z., GRANT, G.A. & GOLDBERG, G.I. (1989). SV40-transformed human lung fibroblasts secrete a 92-kDa type IV collagenase which is identical to that secreted by normal human macrophages. *J. Biol. Chem.*, **264**, 17213–17221.

YAMAGATA, S., TANAKA, R., ITO, Y. & SHIMIZU, S. (1988). Gelatinases of murine metastatic tumor cells. *Biochem. Biophys. Res. Commun.*, **158**, 228–234.

YAMAMOTO, T., KAMATA, N., KAWANO, H., SHIMIZU, R., KUROKI, T., TOYOSHIMA, S., RIKIMARU, K., NOMURA, M., ISHIZUKA, R., PASTAN, I., GAMOU, S. & SHIMIZU, M. (1986). High incidence of amplification of the EGF receptor gene in human squamous carcinoma cell lines. *Cancer Res.*, **46**, 414–416.

YANAGI, H., SASAGURI, Y., SUGAMA, K., MORIMATSU, M. & NAGASE, H. (1991). Production of tissue collagenase (matrix metalloproteinase 1) by human aortic smooth muscle cells in response to platelet-derived growth factor. *Atherosclerosis*, **91**, 207–216.

YANO, H., SHIOZAKI, H., KOBAYASHI, K., YANO, T., TAHARA, H., TAMURA, S. & MORI, T. (1991). Immunohistologic detection of the epidermal growth factor receptor in human eosphageal squamous cell carcinoma. *Cancer*, **67**, 91–98.